# Exploring Ethics: Understanding the Role of Privacy Policies and Institutional Review Boards in Digital Health Companies

**DOI:** 10.2196/70711

**Published:** 2025-05-16

**Authors:** Jacqlyn L Yourell, Kelsey L McAlister, Clare C Beatty, Jennifer L Huberty

**Affiliations:** 1 Fit Minded Inc., PO Box 30271, 2901 E Greenway Road, Phoenix, AZ, 85046, United States, 1 6029356986

**Keywords:** digital health, research, industry, ethics, institutional review board, research ethics committee

## Abstract

Research efforts are growing rapidly in the digital health industry, but with this growth comes increasing ethical challenges. In this viewpoint paper, we leverage over 20 years of combined experience across academia, industry, and digital health to address critical issues related to ethics, specifically privacy policies and institutional review board compliance, which are often misunderstood or misapplied. We examine the purpose of privacy policies and institutional review boards, provide brief examples where companies faced legal and ethical consequences due to shortcomings, and clarify common misconceptions. Finally, we offer recommendations on how digital health companies can improve their ethical practices and ensure compliance in a rapidly evolving landscape.

## Introduction

There has been rapid growth of research initiatives in commercial settings, with the private sector accounting for over 70% of US research and development expenditures in 2017 [1]. Major companies, like Microsoft, Huawei, and Alphabet (Google’s parent company), have significantly expanded their involvement in research, coauthoring academic publications and supporting large-scale projects [2]. Between 2012 and 2021, private sector funding for academic research grew at a faster rate than federal funding [3]. This heightened investment has driven innovation across fields such as digital health, artificial intelligence, and technology.

As private sector investment in research accelerates, concerns are emerging about whether existing ethical oversight mechanisms are evolving sufficiently to address the complexities of commercial research. A recent paper described how studies involving sensitive data, when conducted without formal ethical review, can lead to public misinformation, erode trust in science, and undermine institutional credibility [4]. These concerns are especially relevant in digital health, where existing company practices and policies, such as internal review processes or general user agreements, may not sufficiently address the ethical complexities of data use, highlighting the need for more robust forms of oversight.

Privacy policies and research oversight through institutional review boards (IRBs) have become increasingly important for safeguarding user data. IRBs are committees that review and monitor research involving human participants, aiming to protect participants’ rights and welfare. Trends in ethics for digital health apps are increasingly focusing on data privacy, security, and legal compliance, but ethical concerns are often underemphasized in quality frameworks [5]. For the purpose of this paper, *ethics* refers to the moral guidelines that researchers follow to ensure that they treat participants fairly, respect participants’ rights, and prioritize participants’ well-being in the research process [6]. Considering the frequent misunderstandings and misapplications of ethical principles, particularly those regarding privacy policies and IRBs, the purpose of this paper is to leverage over 20 years of combined experience in academia, industry, and digital health to highlight the importance of complying with ethical practices and integrating these practices in commercial settings. As we discuss privacy policies and IRBs in commercial settings, we focus on their purpose, discuss examples where companies fell short and faced legal and ethical consequences, and clarify common misconceptions. Throughout this paper, we distinguish between what is legally or procedurally required and what constitutes ethical best practices that go beyond compliance to promote transparency, protect participants, and support responsible research in commercial settings. We conclude with recommendations on how digital health companies can enhance ethical practices.

## Privacy Policies

Privacy policies play a critical role in digital health research by establishing the framework for how companies handle user data. However, many of these policies fail to adequately address the ethical complexities surrounding data use for research purposes. Although privacy policies are intended to inform users about how their personal data are collected, stored, and shared, they often lack transparency regarding data use for research [7]. Commercial privacy policies may satisfy baseline legal obligations by disclosing practices like data collection, third-party sharing, and data storage. However, they are not federally required to include language that informs users about how their data may be used to examine user behavior, product effectiveness, or health-related outcomes. As a result, certain safeguards, like obtaining user permission for research or ensuring ethical data use, are not legal requirements; they remain ethical responsibilities that companies are encouraged to uphold [8].

When companies overlook ethical considerations in their privacy policies, the consequences can be severe, ranging from legal issues and public backlash to lasting reputational damage. The consequences of inadequate privacy policies can be seen in several high-profile cases. For example, in the Facebook–Cambridge Analytica scandal, users agreed to their data being shared, as per a privacy policy, but did not fully consent to how that data were sold or used [2]. In this case, Facebook’s privacy policy allowed third-party applications to access users’ data, but users were unaware that their data would be exploited for political profiling, which led to a significant breach of trust and a US $5 billion fine from the Federal Trade Commission [2]. A more recent case involved the meditation app Calm and its use of Twilio—a third-party service—for data collection [9]. Despite Calm’s focus on mental wellness, sensitive information about users’ moods and mental health, which was shared in the app, may have been exposed to other parties through Twilio, highlighting the risks of third-party data handling in digital health contexts [9]. These examples highlight why it is essential for companies to embed and uphold ethical practices at every stage of their operations—starting from product development to data management. These include ensuring transparency, protecting user rights, and maintaining informed consent.

There are common misconceptions about privacy policies that can lead to serious legal and reputational consequences for companies. The first misconception is that companies can share deidentified research data—data from which personal information, such as names or contact details, is removed and data that cannot be linked to an individual—with third parties without informing users. However, when data are originally collected as part of a business and are later used for research, especially if these data include sensitive information, companies may still need to get user permission or have the data reviewed by an ethics team. This review helps with making sure that the data have been properly stripped of identifying details, there is a clear agreement in place about how a third party can use the data, and the data will be stored and handled securely. Even if a company’s privacy policy allows certain uses, relying only on that policy and not taking these extra steps can leave gaps in protection and risk losing user trust. The second misconception is that a privacy policy allows a company to use data for any purpose they choose. A privacy policy typically covers how a company collects, uses, stores, and protects user data, as well as details on data sharing, user rights, and how users can control their information. However, a privacy policy generally does not grant permission to use data for research purposes unless users are explicitly informed and their consent is clearly obtained. A third misconception is that if a company has a clear privacy policy that states that data may be used “for research purposes,” then they do not need additional approval to use the data. Although a privacy policy that clearly outlines information on using data for research can suffice for consent in some cases, IRB approval is still necessary for publishing in peer-reviewed journals; this is for maintaining compliance with privacy protection laws, like the Health Insurance Portability and Accountability Act, and ensuring rigorous ethical oversight (as further detailed in the *Institutional Review Boards* section).

## Institutional Review Boards

Despite their critical role in ensuring ethical practices, IRBs are often underused in commercial settings, leaving gaps in oversight and protection. IRBs review research protocols to confirm that using data, whether already collected or not, does not compromise individuals’ privacy or safety. IRBs ensure that the research design respects participants’ rights, protects individuals, minimizes harm, promotes fairness, and supports responsible research practices [10]. IRB approval is not always legally required for company-led research, particularly when the work is not federally funded or regulated. However, review by an ethics board is recommended when the research involves human participants and is typically required by peer-reviewed journals as a condition for publication [11]. This makes IRB review both an ethical responsibility and a best practice in responsible research. As digital health companies increasingly conduct and publish research that uses their customers’ data, the need for IRB approval becomes more important.

In 2012, Facebook researchers manipulated the news feeds of nearly 700,000 users to study emotional contagion, altering the content to be more positive or negative and tracking emotional responses through language changes [12]. This experiment was conducted without explicit user consent, relying on broad acceptance of Facebook’s general terms of service. Although privacy was not the central concern, this study raised significant ethical questions about informed consent and potential psychological harm to individuals who unknowingly underwent emotional manipulation. As the study lacked IRB oversight, there was no plan to mitigate risk and no consideration to the ethical implications of deceiving users [13]. Although there were no legal consequences, this resulted in increased ethical scrutiny of Facebook’s research practices and changes to its internal review processes, and the study sparked broader discussions about informed consent and corporate responsibility in digital research. Additionally, the journal that published the research—*Proceedings of the National Academy of Sciences*—also published an editorial expression of concern regarding the lack of informed consent in the study [14,15]. Although some might argue that the study posed minimal risk (ie, it was unlikely to harm users), IRB involvement would still have required a structured review of those risks, a clear justification for bypassing consent, and safeguards such as informing users afterward about the study’s purpose and any potential impacts (known as *debriefing*). This case illustrates the real-world consequences of bypassing independent ethical review and highlights how IRBs can proactively address issues of risk, consent, and transparency in digital research.

In another case, researchers scraped and publicly shared sensitive user data from OkCupid, including personal information and responses to private questions, without obtaining user consent or the platform’s approval, thereby violating ethical standards and terms of service [16]. OkCupid filed a Digital Millennium Copyright Act complaint (a legal request to remove copyrighted material), and the data were removed, but the data had already been downloaded nearly 500 times, which leaves concerns about future misuse. These cases illustrate the consequences of bypassing independent ethical review and how IRB involvement could have supported more thoughtful risk assessment and ethical planning. If there had been oversight by an IRB in these cases, the IRB could have flagged these ethical concerns early, enforced standards for data handling and consent, and prevented reputational and ethical fallout.

IRBs are often underused in digital health largely due to misconceptions, similar to what is seen with privacy policies. First, it is often assumed that IRBs are only necessary for academic research and are not necessary for industry-led studies. However, IRBs are an essential part of conducting research that uses human data, regardless of whether the study is initiated in academia or industry, because IRBs ensure that participants’ rights, privacy, and welfare are protected [10]. Second, companies often assume that deidentified data do not need IRB approval. In reality, even when personal information has been removed and the data cannot be linked to individuals, IRB oversight may still be required. IRBs evaluate how the data are anonymized to determine the appropriate level of review needed to protect participant privacy and ensure that research is conducted responsibly. Even when using previously collected deidentified data, IRB review is often recommended to approve the research and ensure that the study minimizes risk and protects people’s privacy. Third, some companies believe that IRB approval slows company progress toward goals. However, IRBs can actually provide flexibility and help to keep costs down by allowing companies to evaluate their products or services more efficiently. For example, companies may submit a broader IRB protocol that covers the data they regularly collect, which can reduce delays when analyzing those data for different research questions. This approach allows companies to use their data for various purposes, including investor presentations and peer-reviewed publications. However, this approach relies on broad consent—a general agreement from users that their data may be used for future research. Although broad consent may satisfy legal standards in some cases, it still raises ethical concerns about whether users fully understand and agree to how their data might be used, especially when the original context of data collection and use was not research-focused [17]. An IRB helps to review whether using data for future research is appropriate by looking at factors like the company privacy policy and how clearly data use was explained to users.

## Recommendations for Digital Health Companies

Herein, we provide recommendations to help digital health companies enhance their ethical practices, streamline processes, and protect consumers. Following these recommendations may ultimately enhance company credibility and ensure compliance with ethical standards in research.

### Recommendation 1: Develop an Ethical Research Framework

We recommend creating clear company policies about how data will be used ethically for research. These policies should explain exactly what data will be collected, how the data will be used, and how the data will be protected. This language can also be used in IRB applications, rather than writing from a blank slate, which can help to accelerate the application process. To ensure the company stays on track, these practices should be regularly reviewed and audited to confirm that they continue to align with the policies and meet ethical standards. This proactive approach helps with maintaining transparency and ensures compliance with legal requirements and recognized standards for the ethical use of human data.

### Recommendation 2: Ensure Clear and Compliant Privacy Policies

Digital health companies should make sure that their privacy policies are accessible; are easy for users to understand; and clearly explain how user data will be used, including use for research purposes. The information should be presented in an up-front manner and not buried in complex legal language. Clear, accessible privacy policies that explain how customer data will be handled and used help to build trust, foster brand preference, and give companies a competitive edge [18]. Having a scientist and/or legal expert review the policies helps with ensuring that they are accurate and meet ethical and legal standards. A clear and accessible privacy policy can help to streamline ethics review by demonstrating that users are made aware of potential research use of their data. In some minimal-risk studies, this can support a request to use the data without requiring individual consent.

### Recommendation 3: Get to Know Commercial IRBs

When selecting a commercial IRB, digital health companies should consider factors like review timelines, costs, and the IRB’s experience with the type of research to be conducted. Some commercial IRBs offer faster review processes at higher costs, while others may be more economical but take longer to complete reviews. We recommend researching the IRB’s reputation and consulting with other companies or professionals who have worked with the IRB, to ensure that the best choice is made for the company’s needs. Although commercial IRBs are a good fit for many companies, it is also worth considering alternative models, such as internal ethics boards, academic partnerships, nonprofit IRBs, or community-based review panels, that may offer advantages depending on factors like independence, transparency, available resources, and the specific nature of the research.

### Recommendation 4: Integrate IRB Processes Into Company Operations and Strategies

To effectively communicate the importance of IRB approval to executives, it is essential to highlight how IRB approval ensures proper oversight of research and data operations, offering a competitive advantage. Digital health companies should integrate IRB processes into their regular workflows (eg, they can develop privacy policies early and have staff who will handle or interact with customer data complete human research protection training). Executives can also be educated about how this ensures consistent ethical practices and smooth approval processes. This proactive approach can help to avoid delays and disruptions in the research cycle. Additionally, one can emphasize how IRB approval contributes to building credibility with investors, partners, and customers. IRB approval can also protect the company from costly legal issues and reputational damage, improve customer loyalty, attract partnerships, and enhance market positioning [18].

### Recommendation 5: Collaborate With Experts

Digital health companies should partner with experienced researchers who understand industry-specific IRB requirements to ensure smooth approval processes. These experts can help with navigating the nuances of commercial IRBs, meeting necessary standards, and avoiding delays and rejections. Their guidance on complex issues can save time and resources while ensuring ethical compliance.

### Recommendation 6: Stay Informed and Adaptable

We also recommend staying current with evolving regulations, industry trends, and best practices in research ethics. As laws and standards around data privacy, informed consent, and IRB processes change over time, it is essential for companies to regularly review and update their internal policies to maintain compliance. This proactive approach may help to prevent legal issues and costly mistakes and demonstrate the company’s commitment to ethical integrity.

## Conclusions

Drawing from over 20 years of combined experience in academia, industry, and digital health, we highlighted a set of critical ethical considerations that commercial research organizations often overlook. In particular, we focused on how privacy policies and IRBs are frequently overlooked in industry, clarifying their functions and benefits for companies. Finally, we provided actionable recommendations on how digital health companies can enhance their ethical practices. By adopting these strategies, companies can strengthen their commitment to ethical research, streamline approval processes, and build trust with both consumers and stakeholders, thereby ultimately driving sustainable growth and innovation in the digital health space.

## Abbreviations

IRB: institutional review board
